# Public Health Word of the Year 2023 — Conflict

**DOI:** 10.1002/puh2.220

**Published:** 2024-07-17

**Authors:** Don Eliseo Lucero‐Prisno, Jerico B. Ogaya, Deborah O. Shomuyiwa, Yusuff Adebayo Adebisi, M. B. N. Kouwenhoven, Isaac Olushola Ogunkola, Odey Goodness, Nafisat Dasola Jimoh, Mohamed Mustaf Ahmed, Thinley Dorji

**Affiliations:** ^1^ London School of Hygiene and Tropical Medicine London UK; ^2^ Department of Medical Technology Institute of Health Sciences and Nursing Far Eastern University Manila Philippines; ^3^ Faculty of Pharmacy University of Lagos Lagos Nigeria; ^4^ University of Glasgow Glasgow UK; ^5^ Department of Physics Xi'an Jiaotong‐Liverpool University Suzhou China; ^6^ YouthRise Dublin Ireland; ^7^ Department of Health Policy London School of Economics and Political Science (LSE) London UK; ^8^ African Young Leaders for Global Health Abuja Nigeria; ^9^ Faculty of Medicine and Health Sciences SIMAD University Mogadishu Somalia; ^10^ Department of Internal Medicine Central Regional Referral Hospital Gelephu Bhutan

**Keywords:** civil war, conflict, humanitarian crisis, political unrest, public health, terrorism, violence

## Abstract

The term “conflict” resonated throughout 2023, echoing prolonged civil wars and heightened global tensions in geopolitical disputes, escalating ongoing rifts among global communities, and exacerbating the humanitarian crisis. This article explores the complex relationship between conflicts and public health, providing a thorough analysis of their dynamics and current prevalence. It aims to illuminate the diverse challenges posed and delineate a holistic path forward to mitigate violent conflicts and ameliorate health disparities, particularly among affected low‐ and middle‐income countries, by advancing an equitable and resilient healthcare system. The expanded perspective of “conflict” reveals far‐reaching consequences that extend beyond borders, significantly straining public health capacity. The world is in dire need of reinvigorating healthcare systems and de‐escalating such violent conflicts due to the relentless exhaustion of resources and the increasing demand for medical emergencies that current responses inadequately address. The damage to vital healthcare facilities in conflict zones severely hampers the provision of necessary and timely medical care, affecting a wide range of health services, including treatment for chronic illnesses, maternal and child care, and mental health support. Additionally, the continuous displacement of people in these areas increases their susceptibility to infectious diseases, raising the possibility of new outbreaks and worsening long‐standing public health challenges. Therefore, prioritizing public health in diplomatic efforts is essential for resolving conflicts and aiding recovery, through building a strong public health strategic approach for a more stable and peaceful global community.

## 1 Introduction

Conflict is universally recognized as both inevitable and a potent force for change and innovation, driving critical advancements and evolution [1]. However, when tensions escalate into violence, they become sources of profound disruption, spreading chaos far beyond their origins as parties embroiled in conflict pursue their aims with little regard for the cost of human life [2]. Despite expectations that 2023 would be a year of global recovery and advancement in public health [3], it was characterized by a significant increase in violent conflicts. The rise in armed conflicts globally has led to humanitarian crises and worsened the Global Peace Index [3]. This rise in both international and non‐international armed conflicts (NIACs) is fracturing communities globally and posing risks to both the environment and human safety [2, 3]. As a result, the concept of “conflict” has become a defining theme of 2023, synonymous with ongoing civil wars, escalating geopolitical tensions, and power struggles that threaten global security and peace.

Currently, the Geneva Academy of International Humanitarian Law and Human Rights oversees more than 110 armed conflicts, with NIACs comprising the majority, accounting for approximately 93% worldwide. These conflicts are distributed across regions: the Middle East and North Africa (>45 armed conflicts), Africa (>35 armed conflicts), Asia (21 armed conflicts), Europe (7 armed conflicts), and Latin America (6 armed conflicts) [4]. Although some conflicts gain widespread attention, others remain less prominent, with durations varying from recent developments to enduring for over five decades. The multifaceted impact of social phenomena on public health is evident, with multilevel conflict assessments revealing interconnected dynamics across personal, community, and nation‐state levels [5].

Civil wars, terrorism, and other forms of armed conflict across different regions significantly undermine healthcare systems’ effectiveness [6]. These conflicts, marked by intense violence, lead to widespread destruction and deep psychological trauma, posing substantial threats to both public health and societal stability [7, 8]. This commentary explores the global impact of conflict on public health, emphasizing its role in exacerbating violence and health disparities, and urging a shift in understanding global health challenges to acknowledge conflicts’ complexity and contradictions.

## 2 The Nexus Between “Conflict” and Public Health

Throughout history, conflicts have been an intrinsic facet of human endeavors, rooted in societal disparities and propelled by antithetical ideologies [7]. Across the ages, global society has borne witness to violent confrontations in various forms, stemming from political, economic, and cultural disparities that have exacted a toll on the lives of many civilians [8]. Although conflicts are often scrutinized through geopolitical lenses, with a focus on political and socioeconomic implications, their impact on public health is equally profound [9]. Conflicts and public health are intertwined, requiring a nuanced understanding of their relationship and an expanded perspective on their catastrophic effects, including health system disruption, disease burden amplification, mental health harm, and healthcare access barriers.

Recent conflicts like the wars in Syria [10], the Russo‐Ukrainian conflict, and the Israeli‐Palestinian dispute [11] have severely violated human rights, exacerbated humanitarian crises [11, 12], and caused widespread trauma, including gender‐based violence, especially against women [13, 14], and harm to children [14, 15]. These conflicts, often rooted in political instability, economic deprivation, and external influences, disrupt social and gender dynamics, impacting broader societal concerns and deepening historical divisions [9, 13, 14]. The Western powers’ historical involvement, exemplified in Gaza's situation, highlights how biased mediation and a focus on geopolitical interests, particularly in Africa and the Middle East [12, 16], have further exacerbated these issues, leading to public health crises, damaged infrastructure, and significant health challenges such as malnutrition, infectious diseases, and mental health disorders [10, 12, 16].

## 3 “Conflict” as a Conundrum: Destructive and Constructive

The term “conflict” embodies a dual nature, capable of inflicting widespread devastation on societies while also serving as a catalyst for transformative change. Conflict escalation, often propelled by enemy propaganda, unfolds as a destructive force, leading to the crippling of healthcare systems, increased disease burden, and erosion of mental well‐being [17]. Deliberate targeting of healthcare facilities and the displacement of healthcare professionals in conflict zones exacerbate challenges faced by affected populations, causing disruptions in health systems, breakdowns of essential medical supply chains, and upsurges in epidemics and starvation [18].

Despite the devastating impacts of conflict, it also presents an opportunity for constructive change in public health. The post‐conflict reconstruction phase provides a critical window to strengthen health systems and address inequalities. Conflicts can unite rival parties over shared health goals, leading to global cooperation and joint efforts in mobilizing resources, improving healthcare delivery, and enhancing capacity [19]. Conflict zones often inspire medical advances, public health policies, and humanitarian endeavors. Understanding how conflict may contribute to positive change is crucial for creating public health policies that capitalize on crises’ potential for growth [20]. This perspective supports a more nuanced view of conflict as both a problem and an opportunity for collaboration and innovation in public health.

## 4 Global Manifestations of Unresolved “Conflict”

The ongoing global surge in conflicts has profoundly shaken the world, giving rise to extremism that not only triggers immediate healthcare exigencies but also imposes a long‐term health burden [18]. The continuous influx of casualties overwhelms healthcare systems, demanding urgent medical interventions. In conflict zones, the provision of emergency medical care becomes an arduous challenge as healthcare facilities bear the brunt of violence, exacerbating an already precarious health situation [18].

Conflicts have wide‐ranging public health consequences beyond immediate physical injuries. One significant issue is the proliferation of drug problems, including trading, production, and use, which often escalate in conflict zones, further burdening public health systems [21]. The instability and lack of governance provide fertile ground for drug trafficking and abuse, which diverts resources from healthcare to military efforts, leading to severe underfunding of health services [9, 22]. Developing and implementing health policy in conflict‐affected countries is a monumental challenge due to the continuous disruption of administrative and institutional structures [23].

According to UNICEF, more than 300,000 severe violations against children in conflict have been confirmed, with about 40% resulting in fatalities over the past decades [24]. Prolonged and unresolved conflicts, like the Syrian war, which was one of the most fatal phenomena for children in 2017 [25], continuously hinder the delivery of healthcare due to the constant destruction of critical health infrastructure. The consequences also affect noncommunicable diseases, maternal and child health, and mental health services [6, 26]. This disruption extends beyond the immediate conflict zone, impacting neighboring countries as refugees depend on their resources and facilities for essential medical care [27], which leads to food insecurity and extreme hunger [18].

Pregnant women in developing countries face increased risks during conflicts, experiencing limited access to prenatal care and skilled delivery assistance, which is further exacerbated by the spread of emerging and reemerging diseases [12, 13, 14]. This results in higher maternal mortality rates and adverse birth outcomes [28]. The healthcare systems are strained and struggle to meet the heightened demand for services, leading to delays in treatment and surgeries, contributing to an increased mortality rate [18]. Extreme violence forces people to seek asylum in nearby countries, exacerbating food insecurity [29], and creating a conducive environment for infectious diseases to spread rapidly [12, 16, 18]. Violent conflicts also lead to a mental health crisis [19]. Continuous exposure to violence, displacement, and loss contributes to widespread psychological trauma. The lack of mental health support services in conflict zones intensifies the mental health burden, leading to long‐term consequences for individuals and communities and creating a silent epidemic that demands urgent attention [20].

## 5 Conflict Resilience‐Building: a Path Forward

Recognizing the dual nature of conflicts, the key to progress lies in navigating toward a more constructive pathway. This involves addressing the immediate challenges conflicts pose to public health while simultaneously harnessing their potential for positive transformation. As conflicts strain health systems, rendering them ill‐equipped to respond to emerging health crises, the pathway forward entails concerted efforts to rebuild and strengthen these essential systems. The international community, collaborating with affected regions, must prioritize the restoration of healthcare infrastructure, the provision of essential services, and the training and deployment of healthcare professionals to ensure a resilient response to health challenges in conflict zones.

To address the increased disease burden in conflict‐affected areas, a holistic approach is necessary. This includes targeted vaccination campaigns, improved access to clean water and sanitation facilities, and innovative strategies to counter the spread of infectious diseases. Multi‐sectoral collaboration involving health organizations, humanitarian efforts, development initiatives, and security sectors is essential to tackle the root causes of disease proliferation and develop public health interventions accordingly. Additionally, interventions should prioritize mental health support services due to the significant psychological impact of conflicts. Community‐based initiatives, psychosocial support programs, and culturally sensitive mental health services can contribute to fostering resilience and promoting well‐being in conflict‐affected populations.

Efforts for recovery must encompass solutions that address physical and financial impediments. Given the vulnerability of healthcare infrastructure during conflicts, strategic planning, resource allocation, and fortifying essential healthcare delivery with innovative technologies are vital for building health system resilience. Strong international coordination through organizations such as the World Health Organization is needed for effective de‐escalation of conflicts through diplomacy and mediation [30]. Rebuilding transportation infrastructure and ensuring financial support for affected populations are critical components integral improving access to healthcare in conflict settings.

## 6 Conclusion

Conflict in 2023 emerged as a critical public health determinant, emphasizing the need for resilient healthcare systems and concerted global efforts to mitigate violence and address its adverse effects. Conflicts not only disrupt healthcare infrastructure and services but also amplify disease burdens, mental health issues, and health disparities. The intricate interplay between conflict and public health underscores the importance of a holistic approach to address both immediate and long‐term health challenges in conflict‐affected regions. Strong measures and sustainable initiatives must focus on ensuring a resilient healthcare system for the safety and well‐being of conflicted populations to deliver adequate essential services, thereby promoting mental health support in the face of ongoing turmoil through the concerted efforts of global authorities. Recognizing and addressing this nexus is crucial for building a more stable and peaceful global community.

## Author Contributions

D.E.L.P. conceptualized the study. J.B.O. prepared the first draft. All authors reviewed and approved the final manuscript.

### Ethics Statement

The authors have nothing to report.

## Conflicts of Interest

Don Eliseo Lucero‐Prisno III and M. B. N. Kouwenhoven are Editorial Board members of Public Health Challenges and are coauthors of this article. Deborah Oluwaseun Shomuyiwa, Thinley Dorji, Goodness Ogeyi Odey, Isaac Olushola Ogunkola, and Yusuff Adebayo Adebisi are Youth Editorial Board members of Public Health Challenges and a coauthor of this article. To minimize bias, they have been excluded from all editorial decision‐making related to the acceptance of this article for publication.

## Data Availability

Data sharing not applicable to this article as no datasets were generated or analyzed during the current study.
